# Analytical Physicochemical and Functional Studies to Compare AryoTrust, a Follow-On Biologics, with the Originator Trastuzumab (Herceptin)

**DOI:** 10.3390/pharmaceutics18030383

**Published:** 2026-03-20

**Authors:** Khalid Kadhem Al-Kinani, Hussein Kadhum Alkufi, Salam Shanta Taher

**Affiliations:** 1 Department of Pharmaceutics, College of Pharmacy, University of Baghdad, Baghdad 10071, Iraq; sallam.hashem@copharm.uobaghdad.edu.iq; 2 Department of Pharmacognosy, College of Pharmacy, University of Thi-Qar, Thi-Qar 64001, Iraq; husseinalkufi21@utq.edu.iq

**Keywords:** trastuzumab biosimilar, analytical comparability, monoclonal antibody characterization, glycosylation, higher-order structure, biological activity

## Abstract

**Background**: Trastuzumab is a blockbuster monoclonal antibody that has revolutionized the treatment of HER2-positive breast and gastric cancers. With the increasing availability of biosimilar monoclonal antibodies in clinical practice, independent verification of biosimilarity using products sampled from a real-world supply chain is important to assure clinicians and the patients to use these products confidently. **Objective**: The aim of this study is to assess the biosimilarity of AryoTrust, a trastuzumab biosimilar, in comparison with the reference product Herceptin. AryoTrust and Herceptin products were randomly withdrawn from Iraqi hospitals to reflect medicines administered in real clinical settings. **Methods**: AryoTrust and Herceptin were compared using an extensive set of orthogonal analytical techniques which included SDS-PAGE, ion-exchange chromatography, capillary isoelectric focusing, peptide mapping, N-glycan profiling, circular dichroism, differential scanning calorimetry, and surface plasmon resonance. In addition to these teste, functional comparability was also tested using an HER2-dependent cell-based proliferation inhibition bioassay. **Results**: The results showed that both products have highly comparable profiles in all assessed attributes. The analysis showed similar molecular integrity and purity, identical primary structure, comparable charge heterogeneity, similar isoelectric points (pI) of the main isoform, close glycosylation patterns (mainly, by core-fucosylated complex-type glycans), similar higher-order structural features, and thermal stability. The receptor binding studies exhibited comparable binding affinities with Fcγ receptors and FcRn. Finally, the cell-based bioassay revealed comparable dose–response curves with similar EC_50_ values and relative potency. **Conclusions**: The integrated analytical and functional data support the biosimilarity of AryoTrust to the reference product Herceptin, which has been marketed and used in Iraq. This study provides real-world scientific evidence supporting confidence in the quality and comparability of this trastuzumab biosimilar and reduces any doubt in the product and at the same time emphasizes the value of independent post-marketing biosimilarity assessments.

## 1. Introduction

Biological medicinal products have transformed the treatment of many serious and life-threatening diseases, including cancer, autoimmune disorders, and inflammatory conditions. Among these, monoclonal antibodies represent one of the most complex and clinically impactful classes of therapeutic agents. Trastuzumab, a humanized monoclonal antibody directed against the human epidermal growth factor receptor 2 (HER2), has significantly improved clinical outcomes in patients with HER2-positive breast and gastric cancers and remains an essential component of modern oncology therapy [1,2].

With the expiration of patents for many originator biological products, the development of biosimilars has emerged as a key strategy to expand patient access to effective biologic therapies while reducing healthcare costs. A biosimilar is defined as a biological medicinal product that is highly similar to an already authorized reference product, notwithstanding minor differences in clinically inactive components. However, unlike small-molecule generics, biosimilars cannot be considered identical to their reference products due to the inherent complexity of biologics and the nature of their manufacturing processes [3].

Biological products are typically large, protein-based molecules with molecular weights far exceeding those of conventional small-molecule drugs. Their size and structural complexity give them what is called higher-order structures which include secondary, tertiary, and in some cases, quaternary structures. The conformation of these higher-order structures is stabilized mainly through weak non-covalent interactions such as hydrogen bonding, electrostatic interactions, and van der Waals forces. As a consequence to this weakness in interactions, protein therapeutics become highly sensitive to changes in the environment and in the manufacturing conditions. Even minor variations in production processes, formulation, or handling may influence critical quality attributes, which could affect stability, biological activity, and clinical performance [4,5,6].

In addition, monoclonal antibodies are usually produced in living systems, mainly mammalian cell lines, and this introduces further complexity to the process and the resultant product. Hence, any variation in cell line characteristics, culture conditions, purification steps, and/or formulation strategies can influence the protein post-translational modifications such as glycosylation, as well as charge heterogeneity and impurity profiles. These factors are significant contributors in batch-to-batch variability and explain why exact replication of the originator biologic (or even different batches of the originator) is not an easy process. As a consequence to this inherent variability in producing protein therapeutics, the regulatory requirement for biosimilars differs fundamentally from those applied to generic small-molecule drugs [7,8,9].

Based on the abovementioned challenges, stringent regulatory authorities such as the European Medicines Agency (EMA) and the U.S. Food and Drug Administration have established dedicated, science-based pathways for approval of biosimilars different than those for generics. These pathways are primarily reliant on the concept of a stepwise comparability exercise and demonstrating of what is called “totality of evidence” requiring demonstration that the proposed biosimilar has no clinically meaningful differences from the reference product in terms of quality, safety, and efficacy. Although minor procedural differences exist between jurisdictions, both EMA and FDA guidelines emphasize that extensive analytical characterization constitutes the most sensitive and discriminatory component of biosimilar development.

In this regulatory paradigm, analytical similarity forms the foundation of the development program and supports the justification for a reduced non-clinical and clinical data package, provided that high similarity is convincingly demonstrated. Therefore, approval of a biosimilar does not necessitate a full independent clinical development program; rather, it relies primarily on robust physicochemical and functional comparability, supplemented by targeted non-clinical and clinical studies when residual uncertainty remains [10,11,12].

The analytical comparability exercise constitutes the foundation on which a biosimilar development program is based. This exercise involves a head-to-head comparison of the proposed biosimilar and the reference product using a wide range of orthogonal and complementary techniques. The exercise typically begins with the evaluation of molecular integrity, purity, and primary structure, as identity at the amino acid sequence level is a prerequisite for biosimilarity and a biosimilar cannot have a different amino acid sequence than the originator. Afterward, analyses that checks post-translational modifications, charge heterogeneity, and glycosylation, characterization of higher-order structure and conformational stability are a must do. Additionally, functional assays, including receptor binding studies and cell-based bioassays, are employed to confirm that structural similarity translates into comparable biological activity [13,14].

The comprehensive analytical characterization offers multiple benefits in the development program. First, it reduces any residual uncertainty regarding possible differences between products, supports the justification for reduced clinical development, and enables the application of an important regulatory principle called extrapolation of indications. In this regard, demonstrating similarity across critical quality attributes using the comparability exercise is not only descriptive but it plays a decisive role in demonstrating and establishing biosimilarity [15,16].

Trastuzumab is a full-length monoclonal antibody which represents a particularly relevant case for biosimilar development due to its widespread clinical use, complex mechanism of action, and well-characterized structure–function relationships. Not only dependent on HER2 binding, trastuzumab’s clinical activity involves immune-mediated mechanisms that rely on the Fc region attributes [17]. Thus, comprehensive physicochemical and functional characterization of these Fc region attributes are essential. Consequently, trastuzumab biosimilars must undergo particularly rigorous analytical evaluation to satisfy the regulatory and clinical expectations of a good alternative choice to healthcare providers [18].

Since biosimilars entered global markets about two decades ago and became increasingly incorporated into routine clinical practice, independent analytical assessments using commercially available products can provide valuable complementary evidence. Such studies contribute to scientific transparency and can help in reinforcing confidence in biosimilars as being therapeutically equivalent products to the originator among clinicians, patients, and regulators, particularly as multiple products may exist at the same time [19,20].

The present study applies a comprehensive and orthogonal analytical and functional characterization platform to evaluate the biosimilarity of AryoTrust to the reference product Herceptin. A set of well-established techniques were employed to assess molecular integrity, primary structure, charge heterogeneity, glycosylation, higher-order structure, receptor binding, and biological activity. The molecular and functional characterization in this work aims to provide a rigorous and internationally relevant demonstration of trastuzumab biosimilarity consistent with current regulatory expectations.

## 2. Materials and Methods

### 2.1. Materials

AryoTrust (trastuzumab; test product) and Herceptin (trastuzumab; reference product), each supplied as 440 mg vials, were used for all comparative analyses. Both products were randomly withdrawn from routine stocks of Iraqi hospital pharmacies as commercially available formulations in unopened and unexpired vials. Prior to analysis, hospital-sourced vials were verified for batch and expiry information, packaging integrity, and compliance with labeled storage conditions (2–8 °C). After reconstitution according to manufacturer instructions, samples were visually inspected for clarity and absence of visible particulates. Only vials meeting these criteria were included in the study.

### 2.2. SDS-PAGE Analyses

Sodium dodecyl sulfate-polyacrylamide gel electrophoresis (SDS-PAGE) was employed to assess the molecular integrity and purity of AryoTrust in comparison with the reference product Herceptin. Analyses were performed under both non-reducing and reducing conditions [21].

Samples were prepared by mixing each product with SDS sample loading buffer. For reducing conditions gel, 2-mercaptoethanol was added to the loading buffer up to a concentration of 5% (*v*/*v*). The prepared samples were then heated to about 90 °C for 7–10 min to ensure complete denaturation of the protein samples for better electrophoresis.

The analysis was carried out using 12% polyacrylamide gels, where amounts of protein (approximately 4 µg per lane) were loaded into the gel wells. A molecular weight marker was also loaded to one of the gel wells to allow prediction of protein band sizes. Electrophoresis was conducted at 180 V for 55 min using standard Bio-Rad protein electrophoresis apparatus.

After the run was completed, the gels were stained with Coomassie Brilliant Blue R-250 and then destained with the destaining buffer to visualize protein bands [22]. The electrophoretic profiles of AryoTrust and Herceptin were compared qualitatively with respect to band number, migration pattern, and apparent molecular weight under both reducing and non-reducing conditions.

### 2.3. Ion-Exchange Chromatography (IEC) for Charge Variant Analysis

Charge variant profiling of AryoTrust and Herceptin was performed using cation-exchange high-performance liquid chromatography (CEX-HPLC). Trastuzumab samples were diluted to a final concentration of 1 mg/mL using mobile phase A. To minimize charge heterogeneity arising from C-terminal lysine residues, samples were enzymatically treated with carboxypeptidase B prior to analysis. Briefly, 300 µL of the antibody solution was mixed with 5 µL of carboxypeptidase B (5 mg/mL, porcine pancreas origin) and incubated at 37 °C for 2 h [22,23].

Following digestion, 80 µL of each sample was injected into a TSKgel CM-STAT cation-exchange column (7 µm, 4.6 × 100 mm) operated on an HPLC system equipped with UV detector and the detection was set at 280 nm. Chromatographic separation was conducted at a flow rate of 0.8 mL/min using a linear salt gradient. Mobile phase A consisted of a phosphate buffer (10 mM, pH as specified in the method), while mobile phase B comprised the same buffer supplemented with 100 mM sodium chloride. Elution was achieved by increasing the proportion of mobile phase B over the course of the run.

The eluted charge variants were classified as acidic, main, or basic species based on their relative retention times. The relative abundances of these variants were calculated by integrating peak areas.

### 2.4. Capillary Isoelectric Focusing (cIEF)

Capillary isoelectric focusing was performed to characterize the charge variants of the two products, AryoTrust and Herceptin [24]. The samples were prepared using the same protein concentration which is approximately 1 mg/mL and using a cIEF sample matrix containing carrier ampholytes which cover the relevant pH range for IgG molecules. The mix also contained additives to ensure stable focusing and appropriate electroosmotic flow control. Synthetic pI markers were included in each run for calibration purposes.

Separations were carried out in a coated fused-silica capillary operated under high-voltage conditions until isoelectric focusing was achieved. Focused protein zones were subsequently mobilized toward the UV detector operated at 280 nm. A calibration curve was generated from the migration times of the used pI markers to allow conversion of analyte migration times to isoelectric point (pI) values.

### 2.5. Reversed-Phase HPLC Peptide Mapping Analysis

Samples of AryoTrust and the reference product Herceptin were subjected to enzymatic digestion to prepare them for peptide mapping analysis. Prior to digestion, samples were adjusted to a concentration of approximately 10 mg/mL and subjected to denaturation using the chaotropic buffer (guanidine hydrochloride, EDTA, and Tris-HCl at neutral pH). Dithiothreitol (DTT) was used to reduce the disulfide bonds in the IgG molecules, followed by incubation at 37 °C for 30 min. Afterward, the free thiol groups were alkylated by treatment with iodoacetamide (IAA) at room temperature in darkness.

The reaction mixture was diluted with a urea-based digestion buffer to reduce denaturant concentration. Enzymatic digestion was then carried out using sequencing-grade trypsin under controlled conditions for about 18 h at room temperature. Finally, trifluoroacetic acid (TFA) was added to stop the digestion.

The resultant peptide mixtures were separated using a C18 analytical column (4.6 × 150 mm, 5 µm particle size, 300 Å pore size) connected to high-performance liquid chromatography (HPLC) where the UV detector was set at 280 nm. Gradient elution was used with aqueous and organic mobile phases containing an acidic modifier to achieve better separation of the peaks.

### 2.6. N-Glycan Profiling

N-linked glycans were enzymatically released from AryoTrust and the reference product Herceptin using PNGase F according to standard procedures. Released glycans were fluorescently labeled and subsequently analyzed by hydrophilic interaction liquid chromatography (HILIC).

Chromatographic separation was performed on a TSKgel Amide-80 column (TOSOH) using a gradient elution system. Glycans were detected by fluorescence with excitation and emission wavelengths set at 360 nm and 425 nm, respectively. Glycan species were identified based on retention times, and the relative abundance of individual N-glycan structures was determined by peak area normalization.

### 2.7. Circular Dichroism (CD) Analysis

Circular dichroism was selected as an orthogonal technique to assess secondary structural elements and detect potential conformational differences between the test and reference products. Because subtle alterations in higher-order structure may affect biological function, far-UV CD provides a sensitive tool for comparing overall folding and secondary structure composition of monoclonal antibodies [25].

Samples of AryoTrust and the reference product Herceptin were prepared for circular dichroism (CD) analysis by buffer exchange to remove formulation excipients, where the samples were transferred into 10 mM potassium phosphate buffer (pH 7.6) using centrifugal ultrafiltration devices with an appropriate molecular weight cutoff. Final protein concentrations were adjusted to 200 μg/mL prior to analysis using the same buffer.

Far-UV CD spectra of the samples were recorded using a Jasco CD spectropolarimeter over a wavelength ranging from 195 to 245 nm at 20 °C, employing a 1 mm path-length quartz cuvette. Scanning was done at a constant scan rate, and three replicate scans were collected for each sample. The averaged spectra were used for analysis following buffer baseline subtraction. Results of CD spectra were expressed as molar ellipticity (deg·cm^2^/dmol) versus wavelength (nm) [26].

### 2.8. Differential Scanning Calorimetry (DSC)

Differential scanning calorimetry was employed to evaluate conformational stability and domain-level unfolding transitions. Thermal transition temperatures (Tm values) are considered sensitive indicators of domain integrity and structural robustness. Comparable thermal profiles between products support similarity in higher-order structural stability, which is a critical quality attribute for monoclonal antibodies [27,28]. First, samples were buffer-exchanged and adjusted to a protein concentration of 1 mg/mL to ensure direct comparability between products and exclude any effect due to formulation excipients.

DSC measurements were performed using a high-sensitivity differential scanning calorimeter. Samples were heated from approximately 40 °C to 95 °C at a controlled scan rate under constant pressure. Corresponding buffer blanks were analyzed under identical conditions and subtracted from sample thermograms.

Thermograms were recorded as heat capacity (Cp) versus temperature, and the thermal transition temperatures (Tm) corresponding to major unfolding events were determined from the maxima of the endothermic transitions. Data analysis was carried out using the instrument’s software, and representative thermograms were used for comparative evaluation of AryoTrust and Herceptin.

### 2.9. Surface Plasmon Resonance (SPR) Analysis

SPR experiments were performed using a Biacore X100 biosensor (GE Healthcare, Chicago, IL, USA) to evaluate the binding interactions between AryoTrust or the reference product Herceptin and recombinant human Fc receptors [29,30]. Sensor chips (CM5) were functionalized using an anti-His antibody capture strategy (His Capture Kit, GE Healthcare). Both active and reference flow cells were activated using a standard amine-coupling protocol with N-ethyl-N′-(3-dimethylaminopropyl) carbodiimide (EDC) and N-hydroxysuccinimide (NHS). Anti-His antibody (50 µg/mL in 10 mM sodium acetate buffer, pH 4.5) was immobilized on the surface, followed by blocking with ethanolamine (pH 8.5). Immobilization levels of approximately 6000–8000 RU were achieved.

His-tagged Fc receptors were captured on the anti-His surface prior to analysis. Binding measurements were carried out using a single-cycle kinetics approach. Serial dilutions of AryoTrust or Herceptin were injected over the receptor surface at a constant flow rate, with defined association and dissociation phases. Experiments were conducted at 25 °C. After each cycle, the sensor surface was regenerated using 10 mM glycine-HCl (pH 1.5).

Sensorgrams were reference-subtracted and analyzed using Biacore Evaluation Software (v2.1). Binding data were fitted to a 1:1 Langmuir interaction model to determine kinetic parameters and equilibrium dissociation constants (KD).

### 2.10. In Vitro Cell-Based Bioassay

The biological activity of trastuzumab was evaluated using an inhibition of cell proliferation bioassay. This assay assesses Fab-mediated binding activity through HER2-dependent growth inhibition. Relative potency of AryoTrust was determined in comparison with the reference product Herceptin.

The human breast cancer cell line BT-474, selected for its high and stable HER2 receptor expression, was used in the assay. Cells were seeded into 96-well microplates at a density of approximately 9000–10,000 cells per well in a volume of 100 μL and allowed to equilibrate at 37 °C for 3 h. Serial dilutions of Herceptin and AryoTrust were prepared and applied to the cells in parallel.

Following addition of test and reference samples, plates were incubated for 90 ± 3 h at 37 °C in a humidified atmosphere containing 5 ± 1% CO_2_. Cell viability and proliferation were quantified using Alamar Blue reagent (Thermo Fisher Scientific, Waltham, MA, USA), which produces a fluorometric signal proportional to cellular metabolic activity. Fluorescence was measured as relative fluorescence units (RFUs) with excitation at 530 nm and emission at 590 nm using a multimode microplate reader (BioTek, Winooski, VT, USA).

Dose–response curves were fitted using a four-parameter logistic (4PL) model under a parallel-line bioassay framework in accordance with United States Pharmacopeia (USP) recommendations for biological assays. Curve fitting and potency estimation were performed using PLA 2.0 software (Stegmann Systems GmbH, Rodgau, Germany). The model assumes parallelism between test and reference dose–response curves. Relative potency was calculated as the ratio of EC_50_ values (reference/test) based on geometric means across independent runs. Ninety-five percent confidence intervals (95% CIs) were generated using the software’s statistical module, and assay validity criteria included evaluation of curve parallelism, slope similarity, and goodness-of-fit parameters prior to acceptance of results.

## 3. Results

### 3.1. SDS-PAGE Analysis

**Figure 1:**
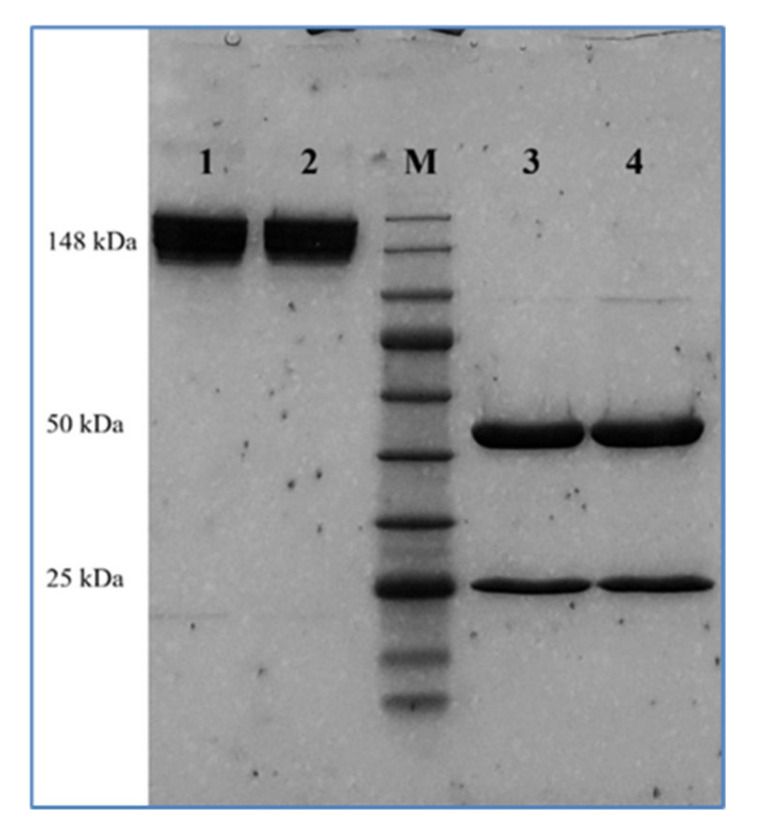
SDS-PAGE analysis of AryoTrust and Herceptin. SDS-PAGE was performed under non-reducing and reducing conditions. Lanes 1 and 2 correspond to AryoTrust and Herceptin, respectively, under non-reducing conditions. Lane M represents the molecular weight marker. Lanes 3 and 4 correspond to AryoTrust and Herceptin, respectively, under reducing conditions in the presence of 2-mercaptoethanol, showing bands corresponding to the heavy chain (~50 kDa) and light chain (~25 kDa).

SDS-PAGE was performed under non-reducing and reducing conditions to compare the molecular integrity and subunit composition of AryoTrust with the reference product Herceptin (Figure 1). Under non-reducing conditions, both products displayed a single dominant band migrating at approximately 148 kDa, consistent with the intact IgG molecule and indicating comparable molecular integrity and purity. No additional high-molecular-weight aggregates or low-molecular-weight fragments were observed for either product.

Under reducing conditions in the presence of 2-mercaptoethanol, both AryoTrust and Herceptin resolved into two major bands corresponding to the heavy chain (~50 kDa) and light chain (~25 kDa). The banding patterns and relative intensities were highly similar between the test and reference products, with no detectable differences in electrophoretic mobility or evidence of degradation products.

### 3.2. Ion-Exchange Chromatography (IEC) Analysis

**Figure 2:**
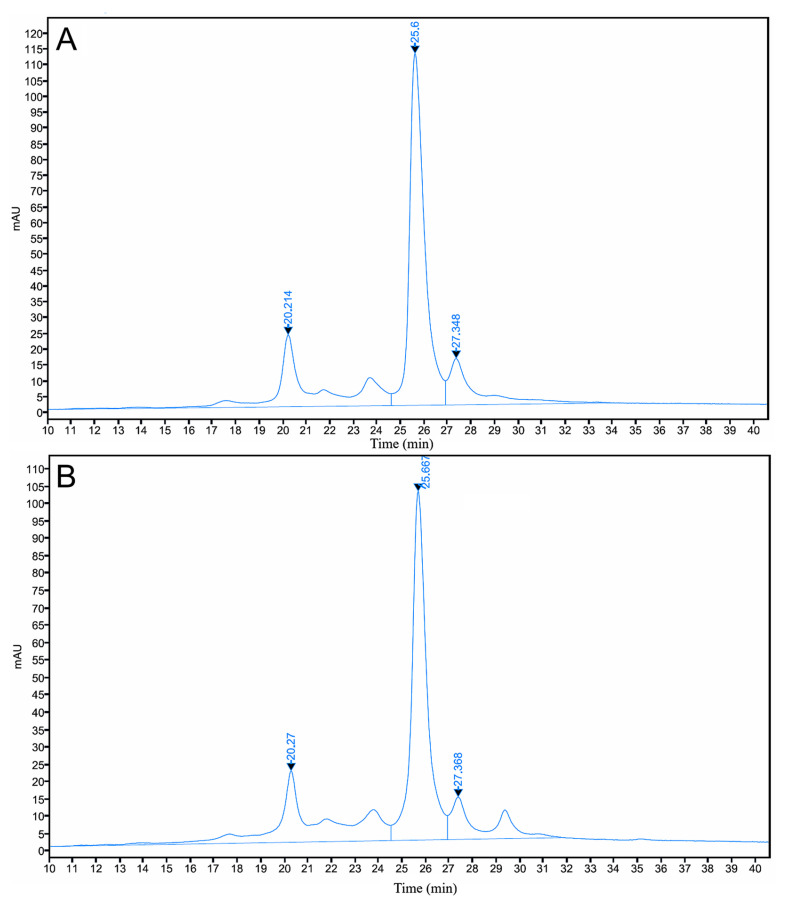

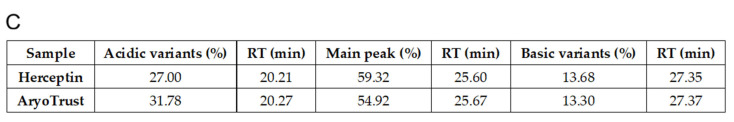
Ion-exchange chromatography (IEC) analysis of charge variants of AryoTrust and Herceptin. Representative IEC chromatograms of Herceptin (**A**) and AryoTrust (**B**) obtained under identical analytical conditions and monitored at 280 nm are shown. Panel (**C**) summarizes the relative peak area (%) and retention times (RTs) of acidic variants, the main peak, and basic variants for each product.

Cation-exchange chromatography profiles of AryoTrust and the reference product Herceptin are shown in Figure 2, and quantitative data are summarized in the table underneath the chromatograms. Both products exhibited chromatograms characterized by multiple resolved peaks corresponding to acidic variants, a dominant main peak, and basic variants.

The elution order and retention times of the major peaks were similar for AryoTrust and Herceptin. The main peak represented the largest proportion of the total chromatographic area for both products. Quantitative analysis based on peak area integration showed comparable relative distributions of acidic, main, and basic variants between AryoTrust and the reference product.

Minor differences in the relative abundance of individual charge variant populations were observed between AryoTrust and Herceptin, while the overall chromatographic profiles and peak distributions remained similar.

### 3.3. Capillary Isoelectric Focusing (cIEF) Analysis

**Figure 3:**
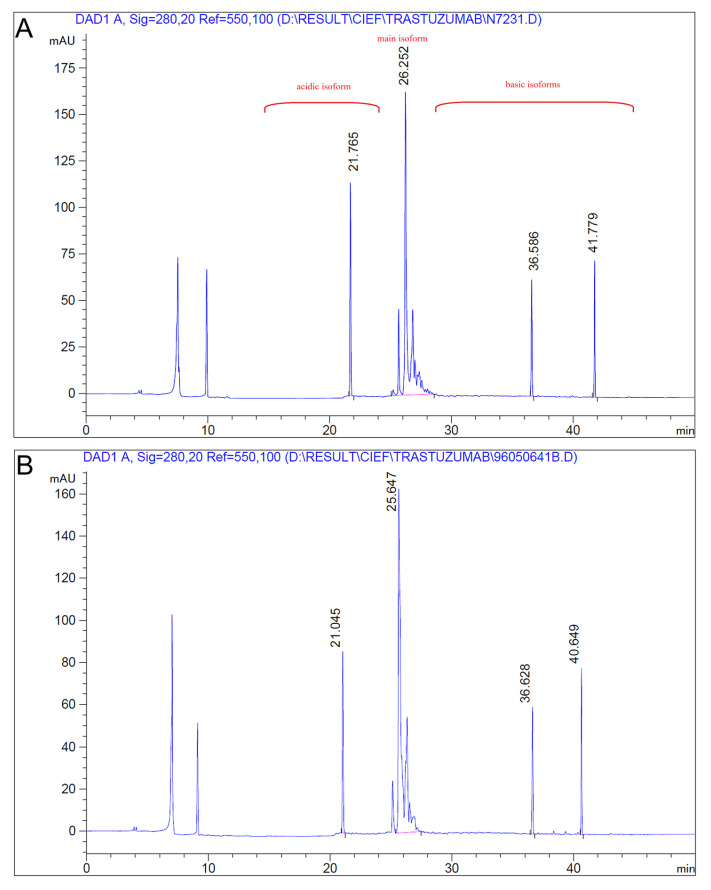
Capillary isoelectric focusing (cIEF) analysis of trastuzumab products. Representative cIEF electropherograms of Herceptin (**A**) and AryoTrust (**B**) obtained under identical analytical conditions are shown. The profiles illustrate the distribution of acidic isoforms, the main isoform, and basic isoforms as indicated.

Capillary isoelectric focusing profiles of AryoTrust and the reference product Herceptin were generated under identical experimental conditions at the same protein concentration. Both products exhibited highly comparable electropherograms (Figure 3), characterized by a dominant main isoform accompanied by minor acidic and basic variants.

Based on migration times calibrated using pI marker standards, the main isoform pI was determined to be 8.7 for both AryoTrust and Herceptin. The coincidence of the main peak position between the two products indicates equivalent isoelectric properties of the principal molecular species. Minor peaks corresponding to charge variants were observed adjacent to the main isoform in both profiles, with similar distribution patterns and relative intensities.

No additional or unexpected isoforms were detected in either product, and no meaningful shift in the main isoform pI was observed between AryoTrust and Herceptin.

### 3.4. Peptide Mapping Analysis

**Figure 4:**
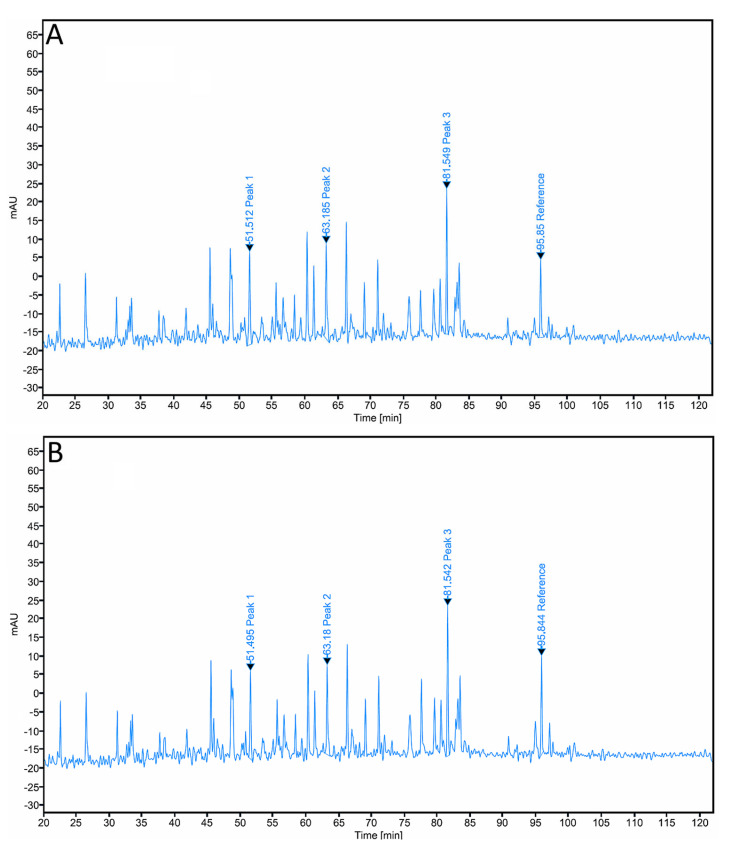
Peptide mapping profiles of trastuzumab products. Representative reversed-phase peptide mapping chromatograms of Herceptin (**A**) and AryoTrust (**B**) obtained after enzymatic digestion (tryptic digestion) and analyzed by RP-HPLC with UV detection under identical analytical conditions are shown.

Peptide mapping profiles of AryoTrust and the reference product Herceptin were obtained following enzymatic digestion and analyzed by reversed-phase HPLC (Figure 4). The chromatograms of both products exhibited comparable peptide elution patterns across the full chromatographic run.

The retention times of the major peptide peaks were closely aligned between AryoTrust and Herceptin. Overlay comparison showed coincident peak positions throughout the chromatograms, with no additional or missing peptide peaks observed for either product. The overall chromatographic profiles demonstrated similar peak distributions and relative intensities under the applied analytical conditions.

### 3.5. N-Glycan Profiling Analysis

**Figure 5:**
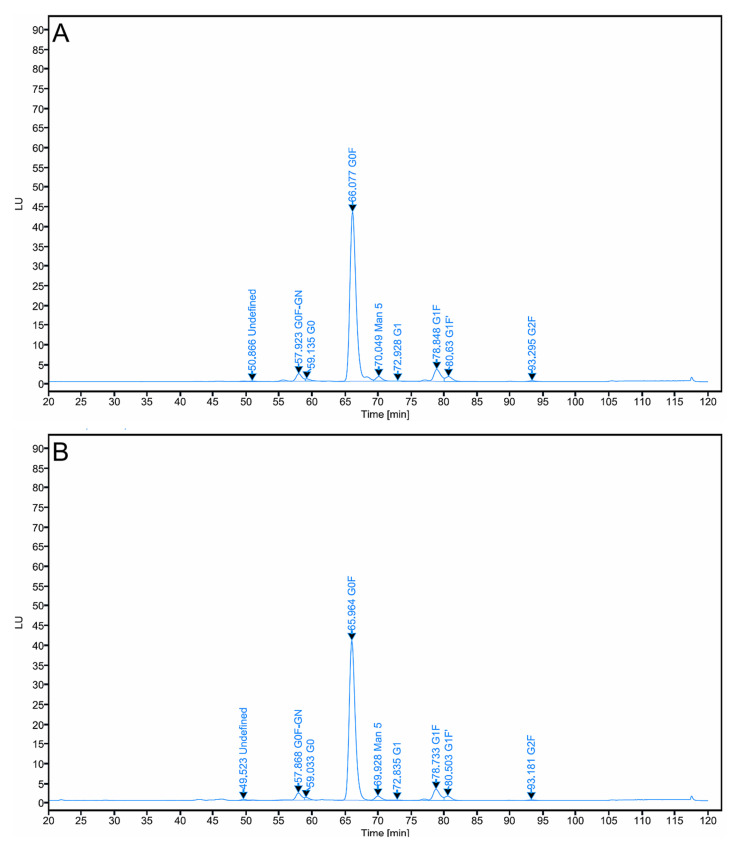
N-glycan profiling of trastuzumab products. Representative fluorescence HPLC chromatograms of released and fluorescently labeled Fc N-glycans from Herceptin (**A**) and AryoTrust (**B**) analyzed under identical chromatographic conditions.

The N-glycan profiles of AryoTrust and the reference product Herceptin were analyzed following enzymatic release and fluorescent labeling of Fc-associated glycans and subsequent HILIC-fluorescence chromatography (Figure 5). Both products exhibited chromatographic profiles characterized by multiple well-resolved peaks corresponding to the major Fc N-glycan species.

The dominant glycan species detected in both AryoTrust and Herceptin were core-fucosylated, complex-type biantennary structures, including G0F, G1F, and G2F. These glycans represented the largest proportion of the total glycan population for both products. In addition, minor glycan species, including high-mannose-type and non-fucosylated glycans, were detected at lower relative abundances in both profiles.

Comparison of retention times demonstrated close alignment of corresponding glycan peaks between AryoTrust and Herceptin, indicating consistent chromatographic behavior under identical analytical conditions. Quantitative analysis based on peak area normalization showed that the relative abundance of individual glycan species was comparable between the test and reference products (Table 1). Small differences in the relative percentages of specific glycan structures were observed; however, the overall distribution patterns of major and minor glycan species were similar between AryoTrust and Herceptin.

No additional glycan peaks unique to either product were detected, and no qualitative differences in glycan composition were observed. The overall N-glycan profiles of AryoTrust and Herceptin were therefore closely matched with respect to glycan identity, retention behavior, and relative abundance.

### 3.6. Circular Dichroism (CD) Analysis

**Figure 6:**
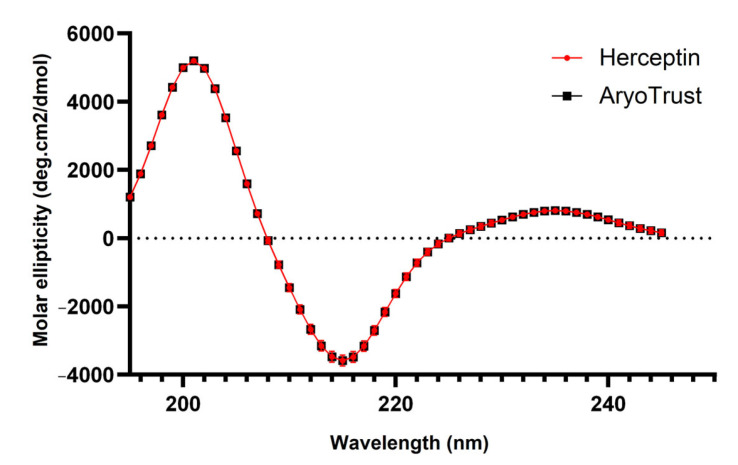
Far-UV circular dichroism (CD) spectra of AryoTrust and Herceptin. Far-UV CD spectra of AryoTrust and the reference product Herceptin recorded over the wavelength range of 195–245 nm are shown. Spectra are presented as molar ellipticity (deg·cm^2^/dmol) as a function of wavelength (nm).

Far-UV circular dichroism spectra of AryoTrust and the reference product Herceptin were recorded over the wavelength range of 200–260 nm under identical experimental conditions (Figure 6). The CD spectra of both products exhibited closely overlapping profiles across the entire spectral range examined.

Both AryoTrust and Herceptin showed characteristic negative ellipticity minima near 208 nm and 222 nm, consistent with the expected secondary structure features of an IgG molecule. The magnitude and shape of the spectra were comparable between the two products, with no detectable shifts in peak positions or changes in overall spectral intensity.

Replicate measurements demonstrated similar spectral reproducibility for AryoTrust and Herceptin. The averaged CD profiles, including associated variability, showed overlapping curves, indicating comparable far-UV CD signatures under the applied conditions.

### 3.7. Differential Scanning Calorimetry (DSC) Analysis

**Figure 7:**
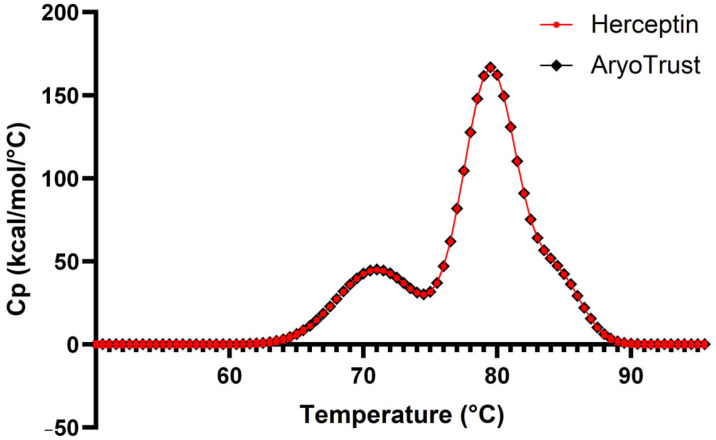
Differential scanning calorimetry (DSC) thermograms of AryoTrust and Herceptin. Representative DSC profiles of AryoTrust and the reference product Herceptin showing heat capacity (Cp) as a function of temperature.

Differential scanning calorimetry thermograms of AryoTrust and the reference product Herceptin are shown in Figure 7, and the corresponding thermal transition temperatures are summarized in Table 2. Both products exhibited three well-defined endothermic transitions within the scanned temperature range.

For Herceptin, thermal transitions were observed with midpoint temperatures (Tm) at approximately 71.6 °C, 82.3 °C, and 90.1 °C. AryoTrust displayed corresponding transitions at approximately 71.4 °C, 82.1 °C, and 89.8 °C, respectively. The differences in the observed Tm values between the test and reference products were small across all detected transitions.

The overall shapes and peak positions of the DSC thermograms were closely matched between AryoTrust and Herceptin. No additional thermal transitions were detected for either product under the applied experimental conditions.

### 3.8. Surface Plasmon Resonance (SPR) Analysis

**Figure 8:**
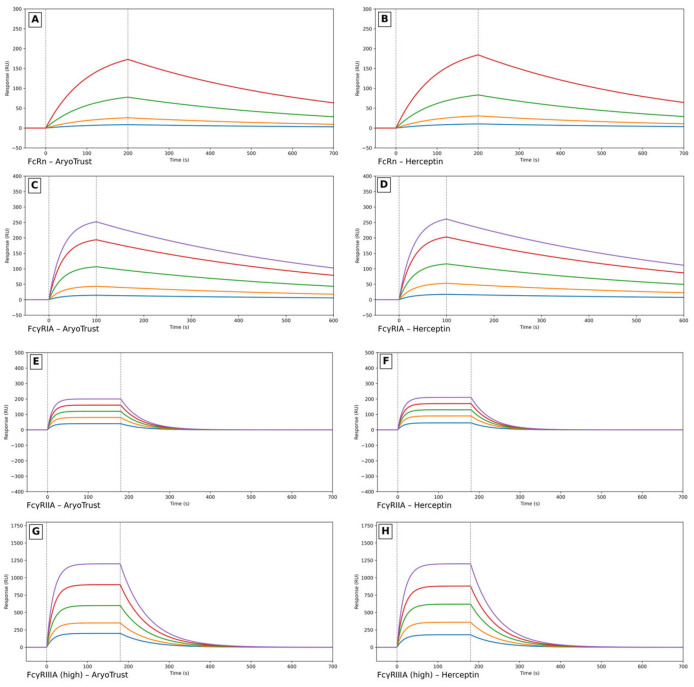
SPR analysis of Fc receptor binding by AryoTrust and Herceptin. Representative surface plasmon resonance (SPR) sensorgrams illustrating the concentration-dependent binding interactions of AryoTrust and the reference product Herceptin with Fc receptors under identical assay conditions at 25 °C. Panels (**A**,**B**) show FcRn binding (AryoTrust and Herceptin, respectively), panels (**C**,**D**) show FcγRIA binding, panels (**E**,**F**) show FcγRIIA binding, and panels (**G**,**H**) show FcγRIIIA binding. Serial dilutions of analytes were injected using a single-cycle kinetics approach. Sensorgrams are reference-subtracted and demonstrate comparable association and dissociation phases between the two products. Binding data were fitted to a 1:1 Langmuir interaction model to determine equilibrium dissociation constants (KD).

Surface plasmon resonance was used to assess the binding interactions of AryoTrust and the reference product Herceptin with Fcγ receptors (FcγRI and FcγRIIa) and the neonatal Fc receptor (FcRn). Representative sensorgrams obtained at multiple analyte concentrations are shown in Figure 8 (panels A–H), and the equilibrium binding parameters are summarized in Table 3.

For FcγRI, both AryoTrust and Herceptin produced concentration-dependent binding responses with comparable sensorgram profiles and response unit (RU) levels across the tested concentration range. The equilibrium dissociation constants (KD) derived from the binding data were similar for the test and reference products.

Comparable binding behavior was also observed for FcγRIIa. AryoTrust and Herceptin generated similar binding responses and sensorgram shapes, and the calculated equilibrium binding affinities were closely matched between the two products.

Binding to FcRn was evaluated under the applied assay conditions, and both AryoTrust and Herceptin exhibited similar concentration-dependent responses. The equilibrium binding affinities obtained for FcRn interaction were comparable between the two products.

Overall, the SPR analysis demonstrated similar equilibrium binding behavior of AryoTrust and the reference product Herceptin toward Fcγ receptors and FcRn under identical experimental conditions.

### 3.9. Cell-Based Bioassay

**Figure 9:**
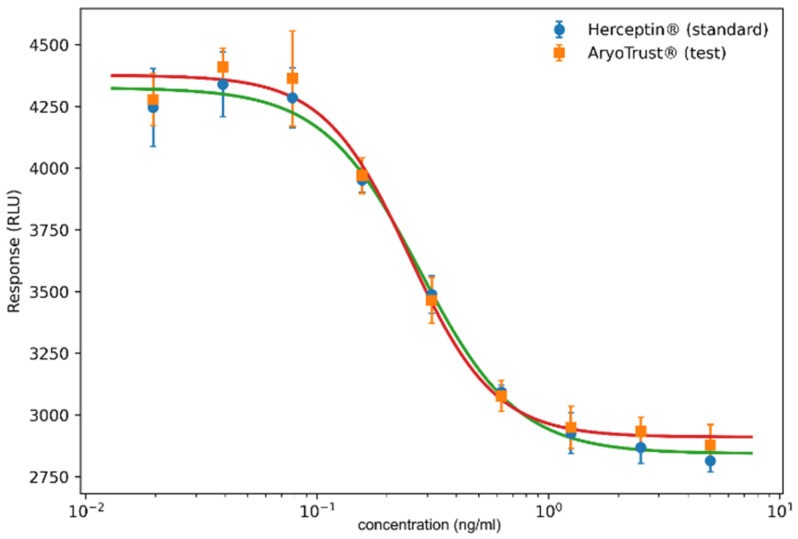
Cell-based bioassay dose–response curves of AryoTrust and Herceptin. Dose–response curves for AryoTrust and the reference product Herceptin obtained using a cell-based quantitative bioassay are shown. Responses were measured as relative light units (RLUs) and plotted against concentration (ng/mL) on a logarithmic scale.

The biological activity of AryoTrust and the reference product Herceptin was evaluated using a quantitative cell-based bioassay (Figure 9), and the corresponding potency parameters are summarized in Table 4. Both products produced concentration-dependent responses over the tested concentration range, yielding sigmoidal dose–response curves.

For Herceptin, the half-maximal effective concentration (EC_50_) values obtained across three independent runs ranged from 0.25 to 0.30 ng/mL, while AryoTrust exhibited EC_50_ values ranging from 0.23 to 0.27 ng/mL. The dose–response curves of AryoTrust and Herceptin showed closely overlapping profiles with comparable slopes and response ranges.

Relative potency values calculated based on EC_50_ ratios were consistent across runs, with individual estimates ranging from 1.06 to 1.17. The geometric mean relative potency of AryoTrust relative to Herceptin was 1.11, with the 95% confidence interval falling within the acceptance limits defined for the assay.

No differences in maximal or minimal response levels were observed between the two products. The overall dose–response behavior and derived potency parameters were comparable for AryoTrust and the reference product under the applied assay conditions.

## 4. Discussion

The increasing availability of biosimilar monoclonal antibodies in routine clinical practice necessitates independent, real-world assessments to ensure that products supplied to patients meet established standards of biosimilarity [20,31]. AryoTrust was developed as a trastuzumab biosimilar with the objective of expanding access to HER2-targeted therapy, particularly in healthcare settings where affordability and resource limitations may restrict access to originator biologics. As a biosimilar, AryoTrust is required to have an identical amino acid sequence to the reference product Herceptin, while any potential differences are limited to manufacturing-related attributes inherent to biologic production processes and must not result in clinically meaningful differences in safety, purity, or potency [32,33].

In this context, the present study evaluated whether AryoTrust and the reference product Herceptin, both commercially available and routinely used within the Iraqi healthcare system, demonstrate comparability when assessed using a comprehensive analytical and functional framework. The two products were randomly withdrawn from Iraqi hospitals and supply channels, thereby representing the actual quality attributes of medicines administered in clinical practice. This market-based approach enhances relevance by addressing biosimilarity at the point of use rather than relying on manufacturer-retained samples and aligns with principles of post-marketing quality surveillance and regulatory assurance in emerging healthcare systems [20,31,34].

The molecular integrity and purity, as assessed by SDS-PAGE analyses under reducing and non-reducing conditions, showed highly comparable electrophoretic profiles for AryoTrust and Herceptin. The presence of a single predominant band, which corresponds to the intact IgG molecule under non-reducing conditions and the expected heavy and light chain bands’ locations under reducing conditions, confirms not only comparable molecular integrity of the two products but also a high degree of purity [35]. The absence of detectable low-molecular-weight fragments or high-molecular-weight species suggests no degradation or aggregation, which is very important given the known impact of such impurities on safety and immunogenicity [36,37].

Primary structure comparability was demonstrated by peptide mapping analysis, which showed highly similar RP-HPLC peptide profiles for both products, AryoTrust and Herceptin. The close alignment of peptide retention times and the absence of additional or any missing peaks between the two product HPLC chromatograms indicate equivalent amino acid sequences and comparable enzymatic digestion patterns. This test is particularly important in light of Anfinsen’s dogma, which states that the primary amino acid sequence contains all the information necessary to determine the native three-dimensional structure of a protein, which is the stable and pharmacologically active structure [38]. Therefore, confirmation of primary structure identity provides a fundamental basis for the observed similarity in higher-order structure and biological function.

Charge heterogeneity, a common feature of monoclonal antibodies arising from post-translational modifications, was evaluated using two orthogonal techniques, ion-exchange chromatography (IEC) and capillary isoelectric focusing (cIEF) [39,40]. Both methods showed comparable distributions of acidic, main, and basic variants for AryoTrust and Herceptin. Also, the cIEF analysis demonstrated identical apparent pI values (pI 8.7) for the main isoform of both products. These results indicate that charge-related modifications such as deamidation, glycation, and C-terminal lysine processing occur to a similar extent in both products and are unlikely to introduce clinically meaningful differences.

Glycosylation represents a critical quality attribute for trastuzumab due to its direct influence on Fc-mediated effector functions and pharmacokinetics. Detailed N-glycan profiling demonstrated that both AryoTrust and Herceptin are dominated by core-fucosylated, complex-type biantennary glycans, primarily G0F, G1F, and G2F, with comparable relative abundances [41,42]. The presence and proportion of fucosylated glycans are particularly important, as core fucosylation is known to modulate binding to FcγRIIIa and, consequently, antibody-dependent cellular cytotoxicity (ADCC) [43]. In addition, the detection of low levels of high-mannose glycans in both products is consistent with reported trastuzumab glycan profiles and is relevant given the association between high-mannose species and increased clearance rates [44]. The overall similarity in glycan composition and distribution therefore supports comparable effector function potential and pharmacokinetic behavior.

Higher-order structural comparability was assessed using circular dichroism (CD) and differential scanning calorimetry (DSC). Far-UV CD spectra of AryoTrust and Herceptin showed overlapping profiles with characteristic minima near 208 nm and 222 nm, reflecting comparable secondary structural elements, including α-helical and β-sheet content typical of IgG molecules. These findings indicate that the folding and secondary structure organization of the two products are closely matched under native conditions [45,46].

DSC analysis provided further insight into conformational stability by revealing three major thermal transitions for both AryoTrust and Herceptin, with closely aligned transition temperatures. These thermal transitions are commonly attributed to sequential unfolding of antibody domains, including the CH2, Fab, and CH3 regions [45]. The close correspondence of transition temperatures suggests similar domain-level stability between the two products. As thermal stability is often correlated with resistance to aggregation and degradation during storage, the observed similarity in DSC profiles supports comparable stability characteristics [47].

Functional comparability was evaluated through surface plasmon resonance (SPR) analysis and a quantitative cell-based bioassay. SPR demonstrated similar equilibrium binding behavior of AryoTrust and Herceptin toward FcγRI, FcγRIIa, and FcRn. Binding to FcRn is particularly relevant for monoclonal antibodies, as it governs IgG recycling and contributes to serum half-life and pharmacokinetic behavior [48]. Comparable FcRn interaction therefore supports similar in vivo exposure profiles. In addition, FcγRIIa binding plays a role in antibody-mediated phagocytosis and immune cell engagement, which are relevant components of trastuzumab’s mechanism of action in cancer therapy. On the other hand, binding to FcγRIIIa triggers antibody-dependent cellular cytotoxicity (ADCC), a central mechanism to the action of trastuzumab [49]. While direct cell-based ADCC, CDC, or ADCP assays were not included in the present study, Fcγ receptor binding analysis—particularly FcγRIIIa interaction—together with comparable glycosylation profiles provides important mechanistic support for similar Fc-mediated effector potential. Additional dedicated effector function assays may be considered in future independent evaluations to further complement the totality-of-evidence framework.

The functional relevance of the observed structural and binding similarities was confirmed using a cell-based bioassay. The cell-based proliferation inhibition assay employed in this study is HER2-dependent and therefore functionally reflects Fab-mediated binding to the HER2 receptor. The comparable dose–response curves and EC_50_ values observed between AryoTrust and Herceptin thus support equivalent HER2-targeted activity under the applied experimental conditions [50]. The consistency of the biological response across multiple assay runs indicates that the cumulative effects of primary structure, glycosylation, higher-order structure, and receptor binding translate into equivalent functional activity at the cellular level [51,52].

The integrated analytical and functional data collectively provide a coherent and comprehensive demonstration of similarity between AryoTrust and the reference product Herceptin. The use of multiple orthogonal techniques increases confidence in the biosimilarity assessment and aligns well with ongoing regulatory expectations for monoclonal antibody biosimilars.

It is important to acknowledge that real-world variability in storage conditions, transportation practices, and handling procedures across healthcare facilities may influence certain quality attributes of biological products over time. In addition, patient-specific factors such as immunogenicity, disease state, and concomitant therapies contribute to clinical variability that cannot be addressed by analytical studies alone [53]. While the present work demonstrates physicochemical and functional comparability at the product level, it does not evaluate clinical outcomes or pharmacovigilance parameters. Therefore, ongoing post-marketing surveillance and pharmacovigilance remain essential components of biosimilar utilization in clinical practice.

## 5. Conclusions

In this study, AryoTrust and the reference product Herceptin, which were randomly withdrawn from the Iraqi pharmaceutical market, were comprehensively compared using an extensive set of analytical, biophysical, and functional methods. The results of the study demonstrated a high degree of similarity between the two products with regard to purity, primary structure, charge heterogeneity, glycosylation, higher-order structure, receptor binding, and biological activity. Overall, these findings support the biosimilarity of AryoTrust to Herceptin as marketed and used in Iraq. This work provides real-world scientific evidence supporting confidence in the quality and comparability of trastuzumab biosimilars available to Iraqi patients and highlights the value of independent post-marketing biosimilarity assessments.

## Figures and Tables

**Table 1:** Relative abundance of major Fc N-glycan species. Relative distribution (%) and retention times (RT) of major Fc-associated N-glycans identified in Herceptin and AryoTrust, determined by peak area integration of fluorescence HPLC chromatograms.

Glycan Species	RT (min)	Herceptin (%)	AryoTrust (%)
G0F	14.52	81.77	81.37
G1F	15.98	6.82	7.16
G1′F	16.74	2.51	2.43
G2F	18.21	0.52	0.51
Man5	12.35	2.48	2.44
GO-GN	13.64	4.26	4.00
GO	11.82	1.10	1.08

**Table 2:** Thermal transition temperatures determined by DSC for Herceptin and AryoTrust. The melting temperatures (Tm) corresponding to the major thermal transitions were determined from DSC thermograms and are presented for AryoTrust and the reference product Herceptin.

Sample	Parameter	Transition Temperature (°C)
Herceptin	Tm1	71.2
	Tm2	80.5
	Tm3	83.4
AryoTrust	Tm1	71.2
	Tm2	80.3
	Tm3	83.6

**Table 3:** Equilibrium dissociation constants (KD) for Fc receptor binding determined by SPR. Equilibrium dissociation constants (KD) for the interaction of Herceptin and AryoTrust with FcRn, FcγRIA, and FcγRIIIA were determined by SPR and are summarized to enable quantitative comparison of binding affinities.

Receptor	Herceptin KD (M)	AryoTrust KD (M)
FcγRIA	6.9 × 10^−9^	5.7 × 10^−9^
FcγRIIA	2.3 × 10^−6^	1.9 × 10^−6^
FcγRIIIA	9.1 × 10^−7^	8.5 × 10^−7^
FcRn	3.2 × 10^−6^	3.1 × 10^−6^

**Table 4:** EC_50_ values and relative potency determined by a cell-based bioassay for Herceptin and AryoTrust. The EC_50_ values corresponding to biological activity were determined from cell-based bioassay dose–response curves and are presented for AryoTrust and the reference product Herceptin, along with relative potency estimates.

Parameter	Herceptin (Reference)	AryoTrust (Test)
EC_50_–Run 1 (ng/mL)	0.251	0.227
EC_50_–Run 2 (ng/mL)	0.300	0.258
EC_50_–Run 3 (ng/mL)	0.283	0.268
Geometric mean EC_50_ (ng/mL)	0.278	0.251
Relative potency (Ref/Test)	-	1.11
95% CI of relative potency	-	1.05–1.17

## Data Availability

The original contributions presented in this study are included in the article. Further inquiries can be directed to the corresponding author.

## Abbreviations

The following abbreviations are used in this manuscript:

HER2	Human Epidermal growth factor Receptor 2
SDS-PAGE	Sodium Dodecyl Sulfate-Polyacrylamide Gel Electrophoresis
FcRn	Neonatal fragment crystallizable receptor
EC_50_	Half-maximal effective concentration
EMA	European Medicines Agency
FDA	U.S. Food and Drug Administration
IEC	Ion-Exchange Chromatography
CEX-HPLC	Cation-exchange high-performance liquid chromatography
cIEF	Capillary Isoelectric Focusing
EDTA	Ethylenediaminetetraacetic acid
IAA	Iodoacetamide
HILIC	Hydrophilic Interaction Liquid Chromatography
CD	Circular Dichroism
DSC	Differential Scanning Calorimetry
SPR	Surface Plasmon Resonance
RFU	Relative fluorescence unit
pI	Isoelectric point

## References

[1] Kalaw San Pascual J.C., Kangsamaksin T. (2025). Biologics, Biosimilars, and Biobetters: Therapeutic Innovations Reshaping Modern Medicine. Adv. Biol..

[2] Costa R.L.B., Czerniecki B.J. (2020). Clinical Development of Immunotherapies for HER2+ Breast Cancer: A Review of HER2-Directed Monoclonal Antibodies and Beyond. NPJ Breast Cancer.

[3] Tinsley P.S.M., Grande C.C., Olson P.K., Plato P.-C.L., Jacobs M.I. (2018). Potential of Biosimilars to Increase Access to Biologics: Considerations for Advanced Practice Providers in Oncology. J. Adv. Pract. Oncol..

[4] Kumar V., Barwal A., Sharma N., Mir D.S., Kumar P., Kumar V. (2024). Therapeutic Proteins: Developments, Progress, Challenges, and Future Perspectives. 3 Biotech.

[5] Rahban M., Ahmad F., Piatyszek M.A., Haertlé T., Saso L., Saboury A.A. (2023). Stabilization Challenges and Aggregation in Protein-Based Therapeutics in the Pharmaceutical Industry. RSC Adv..

[6] Berkowitz S.A., Houde D.J. (2019). The Complexity of Protein Structure and the Challenges It Poses in Developing Biopharmaceuticals. Biophysical Characterization of Proteins in Developing Biopharmaceuticals.

[7] Lee Ventola C. (2013). Biosimilars: Part 1: Proposed Regulatory Criteria for FDA Approval. Pharm. Ther..

[8] Vulto A.G., Jaquez O.A. (2017). The Process Defines the Product: What Really Matters in Biosimilar Design and Production?. Rheumatology.

[9] Liu H., Nowak C., Shao M., Ponniah G., Neill A. (2016). Impact of Cell Culture on Recombinant Monoclonal Antibody Product Heterogeneity. Biotechnol. Prog..

[10] Kirchhoff C.F., Wang X.Z.M., Conlon H.D., Anderson S., Ryan A.M., Bose A. (2017). Biosimilars: Key Regulatory Considerations and Similarity Assessment Tools. Biotechnol. Bioeng..

[11] Wang J., Chow S.C. (2012). On the Regulatory Approval Pathway of Biosimilar Products. Pharmaceuticals.

[12] Al-Kinani K.K., Ibrahim M.J., Al-Zubaidi R.F., Younus M.M., Ramadhan S.H., Kadhim H.J., Challand R. (2020). Iraqi Regulatory Authority Current System and Experience with Biosimilars. Regul. Toxicol. Pharmacol..

[13] Nupur N., Joshi S., Gulliarme D., Rathore A.S. (2022). Analytical Similarity Assessment of Biosimilars: Global Regulatory Landscape, Recent Studies and Major Advancements in Orthogonal Platforms. Front. Bioeng. Biotechnol..

[14] Stüber J.C., Uhland K., Reiter A., Jakob S., Wolschin F. (2024). Comparative Analytical Evaluation of the Proposed Biosimilar FYB206 and Its Reference Medicinal Product Keytruda^®^. Drugs RD.

[15] Stebbing J., Mainwaring P.N., Curigliano G., Pegram M., Latymer M., Bair A.H., Rugo H.S. (2020). Understanding the Role of Comparative Clinical Studies in the Development of Oncology Biosimilars. J. Clin. Oncol..

[16] Tsuruta L.R., Lopes dos Santos M., Moro A.M. (2015). Biosimilars Advancements: Moving on to the Future. Biotechnol. Prog..

[17] Vu T., Claret F.X. (2012). Trastuzumab: Updated Mechanisms of Action and Resistance in Breast Cancer. Front. Oncol..

[18] Xie L., Zhang E., Xu Y., Gao W., Wang L., Xie M.H., Qin P., Lu L., Li S., Shen P. (2020). Demonstrating Analytical Similarity of Trastuzumab Biosimilar HLX02 to Herceptin^®^ with a Panel of Sensitive and Orthogonal Methods Including a Novel FcγRIIIa Affinity Chromatography Technology. BioDrugs.

[19] Mascarenhas-Melo F., Diaz M., Gonçalves M.B.S., Vieira P., Bell V., Viana S., Nunes S., Paiva-Santos A.C., Veiga F. (2024). An Overview of Biosimilars—Development, Quality, Regulatory Issues, and Management in Healthcare. Pharmaceuticals.

[20] Simoens S., Lockhart C.M., Courmier D.F. (2025). What Role for Real-World Evidence in Market Access of Biosimilars?. Front. Pharmacol..

[21] Laemmli U.K. (1970). Cleavage of Structural Proteins during the Assembly of the Head of Bacteriophage T4. Nature.

[22] Al-Kinani K.K., Jassim Z.E., Taher S.S., Hussein A.A. (2021). Comparative Biosimilar Quality Studies between a Rituximab Product and MabThera. J. Adv. Pharm. Educ. Res..

[23] Hsieh M.C., Zhang J., Tang L., Huang C.Y., Shen Y., Matathia A., Qian J., Parekh B.S. (2024). Characterization of the Charge Heterogeneity of a Monoclonal Antibody That Binds to Both Cation Exchange and Anion Exchange Columns under the Same Binding Conditions. Antibodies.

[24] Cao J., Sun W., Gong F., Liu W. (2014). Charge Profiling and Stability Testing of Biosimilar by Capillary Isoelectric Focusing. Electrophoresis.

[25] Oyama T., Suzuki S., Akao K. (2026). ichi Circular Dichroism Spectroscopy in Protein Engineering and Pharmaceutical Development: Applications in Structural Characterization and Quality Assessment. Protein Expr. Purif..

[26] Joshi V., Shivach T., Yadav N., Rathore A.S. (2014). Circular Dichroism Spectroscopy as a Tool for Monitoring Aggregation in Monoclonal Antibody Therapeutics. Anal. Chem..

[27] Majumdar R., Esfandiary R., Bishop S.M., Samra H.S., Middaugh C.R., Volkin D.B., Weis D.D. (2015). Correlations between Changes in Conformational Dynamics and Physical Stability in a Mutant IgG1 MAb Engineered for Extended Serum Half-Life. MAbs.

[28] Durowoju I.B., Bhandal K.S., Hu J., Carpick B., Kirkitadze M. (2017). Differential Scanning Calorimetry—A Method for Assessing the Thermal Stability and Conformation of Protein Antigen. J. Vis. Exp..

[29] Bartusik-Czubek E., Toboła P., Czubek B., Bednarek M., Balcerek J., Pietrucha T., Jaros S. (2021). Modeling of the Biological Activity of Monoclonal Antibodies Based on the Glycosylation Profile. J. Pharm. Sci..

[30] Shibata H., Harazono A., Kiyoshi M., Saito Y., Ishii-Watabe A. (2025). Characterization of Biosimilar Monoclonal Antibodies and Their Reference Products Approved in Japan to Reveal the Quality Characteristics in Post-Approval Phase. BioDrugs.

[31] Liu Y., Wang Y., Wang M., Zhai S., Hou C., Sun F., Jian L. (2025). Evaluating Biosimilars: Safety, Efficacy, and Regulatory Considerations in Clinical Studies. Int. J. Clin. Pharm..

[32] Farmahini Farahani M., Maghzi P., Jafari Aryan N., Payandemehr B., Soni M., Azhdarzadeh M. (2020). A Randomized, Double-Blind, Parallel Pharmacokinetic Study Comparing the Trastuzumab Biosimilar Candidate, AryoTrust^®^, and Reference Trastuzumab in Healthy Subjects. Expert Opin. Investig. Drugs.

[33] Nodehi R.S., Kalantari B., Raafat J., Ansarinejad N., Moazed V., Mortazavizadeh S.M.R., Hosseinzadeh M., Ghaderi B., Jenabian A., Qadyani M. (2022). A Randomized, Double-Blind, Phase III, Non-Inferiority Clinical Trial Comparing the Efficacy and Safety of TA4415V (a Proposed Trastuzumab Biosimilar) and Herceptin (Trastuzumab Reference Product) in HER2-Positive Early-Stage Breast Cancer Patients. BMC Pharmacol. Toxicol..

[34] Kurki P., Barry S., Bourges I., Tsantili P., Wolff-Holz E. (2021). Safety, Immunogenicity and Interchangeability of Biosimilar Monoclonal Antibodies and Fusion Proteins: A Regulatory Perspective. Drugs.

[35] Kirley T.L., Norman A.B. (2018). Unfolding of IgG Domains Detected by Non-Reducing SDS-PAGE. Biochem. Biophys. Res. Commun..

[36] Wang S., Liu A.P., Yan Y., Daly T.J., Li N. (2018). Characterization of Product-Related Low Molecular Weight Impurities in Therapeutic Monoclonal Antibodies Using Hydrophilic Interaction Chromatography Coupled with Mass Spectrometry. J. Pharm. Biomed. Anal..

[37] Moussa E.M., Panchal J.P., Moorthy B.S., Blum J.S., Joubert M.K., Narhi L.O., Topp E.M. (2016). Immunogenicity of Therapeutic Protein Aggregates. J. Pharm. Sci..

[38] Dill K.A., Ozkan S.B., Shell M.S., Weikl T.R. (2008). The Protein Folding Problem. Annu. Rev. Biophys..

[39] Ascione A., Belfiore M., Vesterinen J., Buda M., Holtkamp W., Luciani F. (2024). Charge Heterogeneity of Therapeutic Monoclonal Antibodies by Different CIEF Systems: Views on the Current Situation. MAbs.

[40] Sarin D., Kumar S., Rathore A.S. (2022). Multiattribute Monitoring of Charge-Based Heterogeneity of Recombinant Monoclonal Antibodies Using 2D HIC-WCX-MS. Anal. Chem..

[41] Reusch D., Tejada M.L. (2015). Fc Glycans of Therapeutic Antibodies as Critical Quality Attributes. Glycobiology.

[42] Higel F., Seidl A., Sörgel F., Friess W. (2016). N-Glycosylation Heterogeneity and the Influence on Structure, Function and Pharmacokinetics of Monoclonal Antibodies and Fc Fusion Proteins. Eur. J. Pharm. Biopharm..

[43] Umaña P., Jean-Mairet J., Moudry R., Amstutz H., Bailey J.E. (1999). Engineered Glycoforms of an Antineuroblastoma IgG1 with Optimized Antibody-Dependent Cellular Cytotoxic Activity. Nat. Biotechnol..

[44] Welch J., Ausin C., Brahme N., Lacana E., Ricci S., Schultz-DePalo M. (2023). The Mannose in the Mirror: A Reflection on the Pharmacokinetic Impact of High Mannose Glycans of Monoclonal Antibodies in Biosimilar Development. Clin. Pharmacol. Ther..

[45] Ha S., Wang Y., Rustandi R.R. (2011). Biochemical and Biophysical Characterization of Humanized IgG1 Produced in Pichia Pastoris. MAbs.

[46] Pérez L.M., Rodríguez Taño A.d.l.C., Martín Márquez L.R., Gómez Pérez J.A., Garay A.V., Santana R.B. (2019). Conformational Characterization of a Novel Anti-HER2 Candidate Antibody. PLoS ONE.

[47] Melien R., Garidel P., Hinderberger D., Blech M. (2020). Thermodynamic Unfolding and Aggregation Fingerprints of Monoclonal Antibodies Using Thermal Profiling. Pharm. Res..

[48] Haraya K., Tachibana T. (2023). Translational Approach for Predicting Human Pharmacokinetics of Engineered Therapeutic Monoclonal Antibodies with Increased FcRn-Binding Mutations. BioDrugs.

[49] Musolino A., Gradishar W.J., Rugo H.S., Nordstrom J.L., Rock E.P., Arnaldez F., Pegram M.D. (2022). Role of Fcγreceptors in HER2-Targeted Breast Cancer Therapy. J. Immunother. Cancer.

[50] Bharali P., Chand S., Chander H. (2025). A Cost-Effective and Robust Cell-Based Bioassay Method for Evaluating the Bioactivity of Trastuzumab-like Antibodies. Biomedicines.

[51] Hutterer K.M., Ip A., Kuhns S., Cao S., Wikström M., Liu J. (2021). Analytical Similarity Assessment of ABP 959 in Comparison with Eculizumab Reference Product. BioDrugs.

[52] Hossler P., Khattak S.F., Li Z.J. (2009). Optimal and Consistent Protein Glycosylation in Mammalian Cell Culture. Glycobiology.

[53] Cappelletto E., Kwok S.C., Sorret L., Fuentes N., Medina A.M., Burleigh S., Fast J., Mackenzie I.S., Fureby A.M., Paulsson M. (2024). Impact of Post Manufacturing Handling of Protein-Based Biologic Drugs on Product Quality and User Centricity. J. Pharm. Sci..

